# Evaluating the therapeutic efficacy of the Chinese herbal medicine Yishen Tongbi decoction in patients with active rheumatoid arthritis: protocol for a randomized, controlled, noninferiority trial

**DOI:** 10.1186/s13063-019-4005-0

**Published:** 2019-12-30

**Authors:** Lianyu Zhao, Meilin Li, Yanping Xu, Lijuan Liu, Mingying Zhang, Guangxing Chen

**Affiliations:** 1 The First Clinical Medical College of Guangzhou University of Chinese Medicine, 12 Airport Road, Baiyun District, Guangzhou, China; 2 Division of Rheumatology and Clinical Immunology, The First Affiliated Hospital of Guangzhou University of Chinese Medicine, 16 Airport Road, Baiyun District, Guangzhou, China

**Keywords:** Yishen Tongbi decoction, Rheumatoid arthritis, Efficacy, Methotrexate, Double-blind, Prospective, Randomized controlled, Traditional Chinese medicine

## Abstract

**Background:**

Rheumatoid arthritis (RA) is a common chronic autoimmune disease that seriously affects the quality of life of patients because of damage to joints. Presently, RA is mainly treated with disease-modifying antirheumatic drugs (DMARDs) or biological agents; however, they offer limited efficacy in some patients. Therefore, additional therapeutic strategies need to be developed. Yishen Tongbi decoction is a traditional Chinese medicine formulation widely used to treat RA in China. However, currently, there is insufficient evidence to recommend its use for the treatment of RA. Therefore, we aim to verify the efficacy of Yishen Tongbi decoction to treat RA by a noninferiority trial, and to provide a basis for its use with a full-scale clinical trial.

**Methods/design:**

One hundred eligible patients with RA will be randomized into two groups of 50 patients. One group will receive Yishen Tongbi decoction and placebo replacing methotrexate (MTX), while the other group will receive MTX and placebo replacing Yishen Tongbi decoction. Patient’s whose visual analogue scale score for pain is greater than 40 mm will be administered nonsteroidal anti-inflammatory drugs (such as enteric-coated diclofenac sodium, 25 mg three times a day); administration of all medications will be recorded. The clinical indicators of patients and their disease activity will be assessed at baseline and at 4, 12 and 24 weeks after treatment initiation. The primary outcome of efficacy will be the proportion of patients who demonstrate a favourable response based on their Clinical Disease Activity Index score at 24 weeks after treatment. All adverse events will be reported.

**Discussion:**

Traditional Chinese medicine theory and modern western medicine research have identified the efficacy of Yishen Tongbi decoction to treat RA. Previous clinical observation and efficacy trials of Yishen Tongbi decoction in animal models for the treatment of RA has demonstrated significant effect. Because of the potential benefits of Yishen Tongbi decoction in the treatment of patients with RA, we designed this double-blind, prospective, randomized controlled trial; the results and conclusions of the trail will be published after the completion of the study.

**Trial registration:**

Chinese Clinical Trials Registry, ChiCTR1900024902. Registered on 3 August 2019.

## Background

Rheumatoid arthritis (RA) is a chronic autoimmune disease that progressively destroys joints and negatively influences other physiological areas and aspects, such as the blood vessels and metabolism. RA affects 1% of the global population, and may occur at any age, with women often being more susceptible to developing RA than men [1]. RA is a chronic disease, and affected individuals need regular treatment for prolonged durations which puts a heavy burden on them [2]. RA not only increases the medical expenses of affected individuals but also leads to functional disability, reduces their ability to work and creates a socioeconomic burden [3]. Therefore, early diagnosis, prompt treatment, and development of new treatment modalities to control inflammation and prevent additional damage is warranted in cases of RA [4].

Disease-modifying antirheumatic drugs (DMARDs) remain the most commonly used therapeutics for RA, and the use of methotrexate (MTX) is preferred over other DMARDs [5]. DMARDs have a limited role in preventing the progression of RA and have potential risks, including infection, malignancy, and production of autoantibodies. Therefore, it is necessary to develop newer antirheumatic drugs with higher efficiency and lower toxicity [6].

Yishen Tongbi decoction is a traditional Chinese medicine (TCM) formulation widely used to treat RA in China. This TCM decoction is made from *Fructus Ligustri Lucidi*, *Herba ecliptae*, *Eucommia ulmoides Oliver*, *Fructus lycii*, *Salvia miltiorrhiza Bge*, and *Tripterygium hypoglaucum (Levl.) Hutch.* In China, *Tripterygium hypoglaucum (Levl.) Hutch* is widely used as a TCM for the treatment of RA. Kunmingshanhaitang tablets which are extracted from *Tripterygium hypoglaucum (Levl.) Hutch* are approved for the treatment of RA by Chinese Pharmacopoeia.

We previously examined the efficacy of Yishen Tongbi decoction on an animal model of RA. The results showed that, compared with MTX alone, *Tripterygium hypoglaucum (Levl.) Hutch* alone, and several other TCM compounds, Yishen Tongbi decoction demonstrated a better effect in improving inflammation, inhibiting synovial hyperplasia, and inhibiting joint destruction [7].

Additionally, we conducted a clinical observational study on the use of Yishen Tongbi decoction to treat RA. By comparing the disease activity and clinical indicators of 30 patients with active RA before and after treatment with Yishen Tongbi decoction, we observed significant improvement in clinical symptoms, inflammation index, and disease activity after treatment with Yishen Tongbi decoction [7].

Thus far there has been little research on the effectiveness of TCM decoction in the treatment of RA. Through this clinical study, we aim to explore the efficacy of Yishen Tongbi decoction in the treatment of active RA and to compare the efficacy of Yishen Tongbi decoction and MTX.

We designed this study as a double-blind, prospective, randomized controlled trial. Our research plan and informed consent were submitted to the Ethics Committee of the First Clinical Medical College of Guangzhou University of Traditional Chinese Medicine in November 2018. After two revisions, ethical approval was obtained on 10 April 2019. The results will be released after the trial is completed.

## Methods

### Study design

**Fig. 1:**
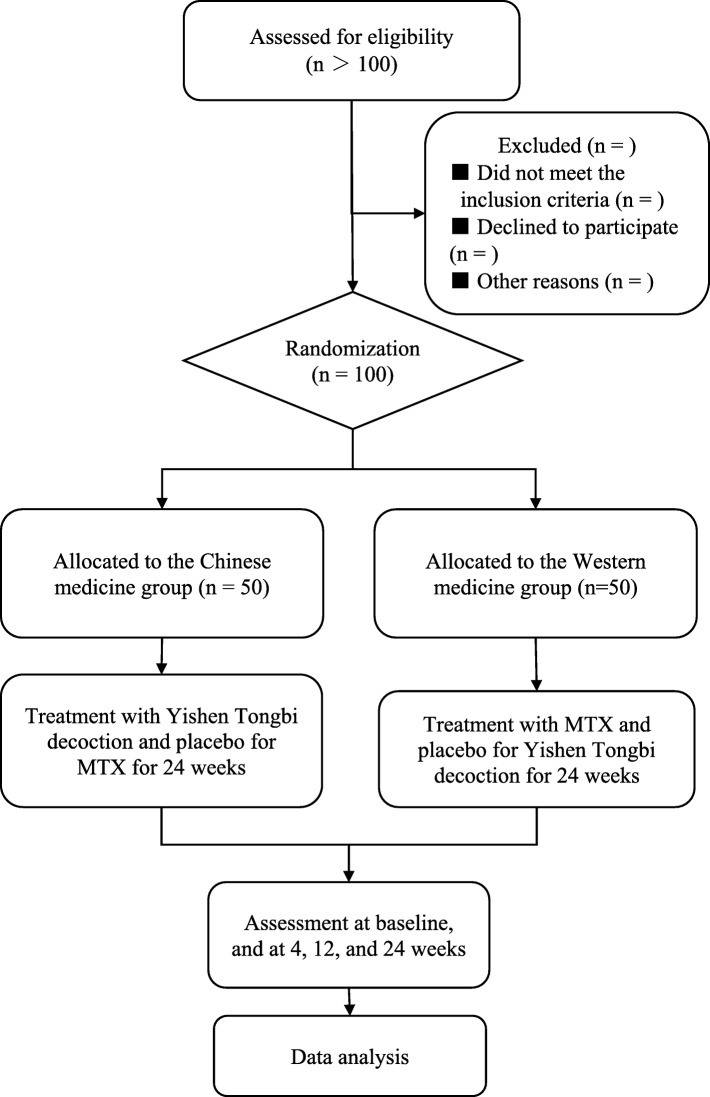
Study flowchart. MTX methotrexate

**Fig. 2:**
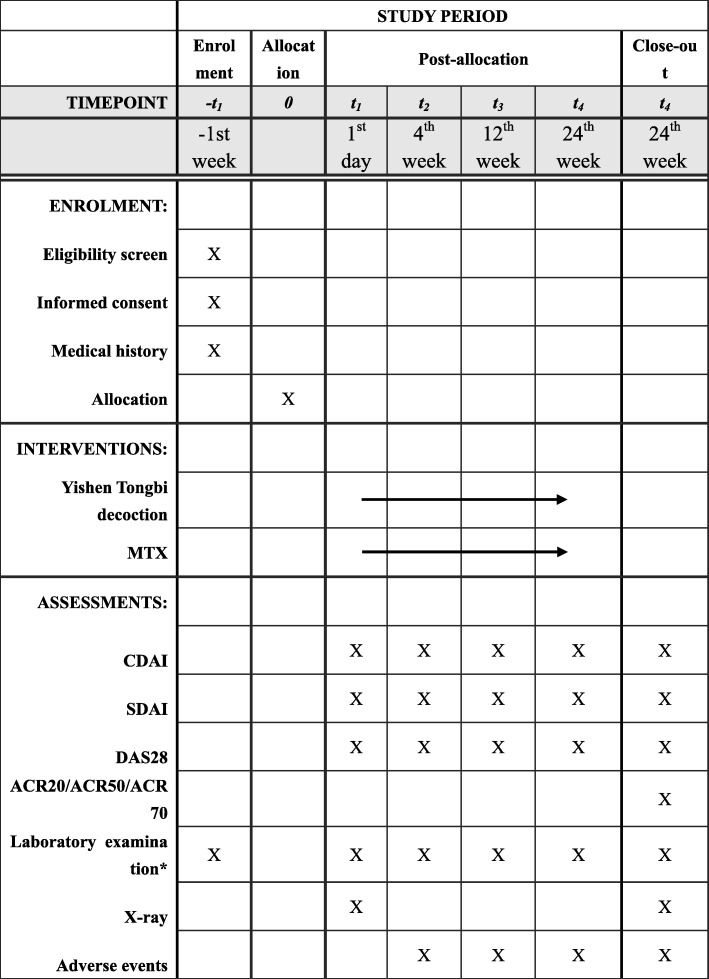
SPIRIT figure showing time points for enrolment, interventions and assessment. *Laboratory examination includes complete blood count, rheumatoid factor, Anticyclic citrullinated peptide antibody, C-reactive protein, erythrocyte sedimentation rate, liver function tests (aspartate aminotransferase, alanine aminotransferase) and renal function (creatinine, urea). ACR20/50/70 improvement in American College of Rheumatology criteria of 20%/50%/70%, CDAI Clinical Disease Activity Index, DAS28 Disease Activity Score in 28 joints, MTX methotrexate, SDAI Simplified Disease Activity Index

This study is a double-blind, randomized controlled, noninferiority trial. One hundred eligible patients with RA will be randomized into two groups of 50 patients. One group will receive Yishen Tongbi decoction and placebo replacing MTX, while the other group will receive MTX and placebo replacing Yishen Tongbi decoction. The study protocol has been approved by the Ethics Committee of the First Affiliated Hospital of Guangzhou University of Traditional Chinese Medicine (approval number ZYYECK-2018-141) and registered with the Chinese Clinical Trial Registry (ChiCTR-1,900,024,902). The study flowchart is shown in Fig. 1. The Standard Protocol Items: Recommendations for Interventional Trials (SPIRIT) checklist is presented in Additional file 1, and the SPIRIT figure is shown in Fig. 2.

### Inclusion criteria

Subjects must meet all of the following requirements:

1. Meet the 2010 American College of Rheumatology (ACR)/European League against Rheumatism classification criteria and must have active RA for at least 6 weeks
2. Be 18–65 years of age
3. Have at least three swollen joints and five tender joints
4. Have C-reactive protein >20 mg/L or erythrocyte sedimentation rate >28 mm/h
5. Provide signed informed consent form

### Exclusion criteria

Subject who do not meet any of the following will be excluded:

1. Pregnant or lactating women
2. Fertility requirements at present or in the future
3. History of severe chronic infection, current infection, and malignant tumour
4. Patients with severe primary diseases, such as those of the cardiovascular, cerebrovascular, hepatic, renal, and hematopoietic systems, and mental illness.
5. White blood cell count <3.5 × 10^9^/L, haemoglobin <100 g/L, alanine aminotransferase or aspartate aminotransferase ≥1.5 times the upper limit of normal, serum creatinine greater than the upper limit of normal
6. History of drug allergy
7. Use of DMARDs or other biological agents within the first 3 months of enrolment
8. The investigator believes that it is not appropriate for the patient to participate in this clinical trial

### Randomization and blinding

A random sequence of 100 participants from the First Affiliated Hospital of Guangzhou University of Chinese Medicine was generated by computer software (Excel) and, thereafter, segmented randomly. We set 25 blocks, each of which included four participants. Two participants of the four were randomly assigned to the Chinese medicine group and two to the western medicine group. The blinded results are kept opaque envelopes; one is kept by the research sponsor and the other by the pharmacy leader. Both researchers and patients are blinded to the grouping. Unblinding will be carried out before the statistical analysis, which will be performed by the Professor of Statistics of Guangzhou University of Traditional Chinese Medicine who will also supervise the scene and report the unblinding time, the compliance with the regulations, and the acceptability of the sealed envelopes in the statistical report.

### Interventions

For the TCM (Yishen Tongbi decoction), a dose decoction made from *Fructus Ligustri Lucidi* 15 g, *Herba ecliptae* 15 g, *Eucommia ulmoides Oliver* 15 g, *Fructus lycii* 15 g, *Salvia miltiorrhiza Bge* 15 g, *and Tripterygium hypoglaucum (Levl.) Hutch* 25 g will be used.

For the western medicine, MTX tablets are provided by Shanghai Xinyi Pharmaceutical Co., Ltd.

For the TCM replacement of Yishen Tongbi decoction with placebo, 300 doses of decoctions will be made from water 4.5 L, maltodextrin 200 g, caramel colouring 35 g, sucrose octa-acetate 1.6 g, and liquorice essence 9 g.

For the placebo to replace MTX, 700,000 tablets will be made from Titanium Aluminium Lake 0.607 kg, corn starch 37.8 kg, sucrose 16.8 kg, dextrin 1.68 kg, and magnesium stearate 0.56 kg.

Patients in the Chinese medicine therapy group will be given Yishen Tongbi decoction (150 mL once a day) along with placebo for MTX (starting from 7.5 mg once a week, increasing to 15 mg once a week within 4 weeks). Participants in the western medicine group will be given MTX (starting from 7.5 mg once a week, increasing to 15 mg once a week within 4 weeks) along with placebo for Yishen Tongbi decoction (150 mL once a day). The entire treatment process will last for 24 weeks.

Folic acid tablets (10 mg) will be administered the day after every MTX (or placebo for MTX) treatment. Systematic evaluation shows that the folic acid supplementation during methotrexate treatment can reduce the adverse reactions, such as gastrointestinal side effects and liver damage [8].

Nonsteroidal anti-inflammatory drugs (diclofenac sodium as enteric-coated tablets, 25 mg three times a day) may be added if necessary (for patients with visual analogue scale score for pain ≥40 mm) during the treatment, and such events will be recorded. Treatment can be maintained if the patient has taken a steady dose of a corticosteroid or a nonsteroidal anti-inflammatory drug before the trial. Participants’ medication information will be recorded in the case report form (CRF) at each follow-up visit.

### Outcomes and measurements

This is a double-blind study where neither the researcher nor the patient is aware of the groupings. The investigators will collect the clinical data and assess disease activity at baseline and at 4, 12 and 24 weeks after treatment initiation. Adverse events will be reported in a timely manner.

The primary outcome of efficacy is the proportion of patients who achieve a result of good response on the Clinical Disease Activity Index (CDAI; defined as achieving ≥50% improvement in the CDAI or CDAI ≤2.8) at 24 weeks [9–12].

Secondary outcomes of efficacy are the proportion of patients achieving a result of good response on the Simplified Disease Activity Index (SDAI; defined as achieving ≥50% improvement in the SDAI or SDAI ≤3.3), and those achieving a 20%, 50% and 70% improvement in the ACR criteria (ACR20, ACR50 and ACR70, respectively) at 24 weeks.

### Safety outcomes

At baseline and at each study visit, safety checks will be performed on the patients by evaluating their complete blood count (CBC), aspartate aminotransferase, alanine aminotransferase, creatinine, and urea levels. We will compare the safety of traditional Chinese and western medicines by comparing the abnormalities among the safety indicators of the two groups.

### Adverse event reporting

An adverse event (AE) is the presence or worsening of any syndrome, symptom or disease that occurs in a patient during the clinical trial and which affects the patients’ health. The term AE does not imply a causal relationship with a test drug. The investigators will record the AEs in a timely manner, and signed and dated reports will be submitted to the sponsors, ethics committees, and drug regulatory authorities as required.

Serious AEs refer to events that require hospitalization, prolonged hospital stay, disability (except for joint damage caused by rheumatoid arthritis), an inability to work (except for joint damage caused by rheumatoid arthritis), or which are life-threatening or can cause death during the clinical trial. Incidences of serious AEs among the patients will be promptly notified to the principal investigator by the researcher for appropriate course of action according to the study plan and the project’s standard operating procedure, and will be reported to the Good Clinical Practice office within 24 h and also to the relevant department. We will provide appropriate compensation for any injured subjects.

### Moral consideration

This trial has been approved by the Research Ethics Committee, the First Clinical Medical College of Guangzhou University of TCM, on 10 April 2019. All patients must sign an informed consent form before participating in the study.

### Quality assurance

The personnel participating in the clinical trial will be relatively permanent and will carefully study and discuss the clinical trial plan and manual, and unify the record method and judgment criteria. Researchers will use a pen or gel pen to fill in the CRF in a realistic, detailed, and serious manner. All observations and findings in the clinical trial will be verified to ensure reliability of data and to ensure that the conclusions from the trial are derived from raw data. There are corresponding data management measures in the clinical trial and data processing stages. Researchers will actively take measures to control the case drop-out rate to within 10%. In the event of a drop-out, the investigator should contact the participant, ask for reasons, record the last medication time, and complete the assessment project as far as possible. Laboratory data will be recorded in the CRF, and the original report will be attached with the medical record. Normal ranges of various laboratory parameters will also be recorded; data that is significantly higher or outside the clinically acceptable range will be verified and the necessary instructions will be provided by the physician participating in the clinical trial. The interventional drugs used in the clinical trial will be regularly inspected by the person responsible for administering the drug, and the status of the drug will be accurately recorded. Yishen Tongbi decoction is provided by the department of decoction of TCM in our hospital. Each batch of TCM decoction will be tested by high-performance liquid chromatography to ensure its compliance with quality requirements.

### Data management

The data monitoring committee is composed of the person in charge, data managers, data ombudsmen, and statistical analysts. Data from the CRF table will be entered into the ResMan database by the data administrator using two entry methods. The Ombudsman will check each item in the database, report inconsistent outcome values, and verify each item of the original questionnaire, and correct it if necessary. All research-related data will be kept in the Department of Rheumatology, the First Affiliated Hospital of Guangzhou University of TCM. The personal information of all the study participants will be kept strictly confidential.

### Sample size

A noninferiority test of Yishen Tongbi decoction and MTX will be adopted. The main efficacy end point is good response on the CDAI (improvement ≥50% or score ≤2.8) after 24 weeks. The study was designed to have a power of approximately 80% and a one-sided level of significance of 0.05 (α = 0.05, β = 0.2). The rate reaching the end point of the Yishen Tongbi decoction group was 68.0% from a previous study by our group, and the rate of the MTX group was 52.2% taken from the literature [7, 12]. The noninferiority margin was set to −0.10 [13]. The proportion of cases between the experimental and control groups was set to 1:1. The sample size for each group can be estimated as 45. Considering a drop-out rate of 10%, a total sample size of 100 participants is required. We used the calculation tool at https://www.cnstat.org/samplesize/.

### Statistical analyses

The efficacy of Yishen Tongbi decoction and MTX will be compared by a noninferiority test. We will conduct intention-to-treat and per-protocol analysis on the main results from the trial. The intention-to-treat analysis will analyse all eligible patients who have received at least one dose of medication. The per-protocol analysis will analyse all patients who meet the inclusion criteria and who do not meet the exclusion criteria and who complete the treatment plan. The proportion of subjects achieving good response on CDAI, SDAI and ACR in both groups will be compared. If the good response rate of CDAI in the Chinese medicine group is not lower than the good response rate of CDAI in the western medicine group by a margin of −0.1, it will be declared as noninferiority. The changes in CDAI, SDAI and Disease Activity Score in 28 joints from baseline after the corresponding treatment will be calculated and the results of the two groups will be compared using an independent sample analysis of covariance (ANCOVA). The amount of improvement of various ACR parameters (tender joint count, swollen joint count, visual analogue score, erythrocyte sedimentation rate, C-reactive protein, etc.) will be analysed and ANCOVA will be performed. For safety, the incidence of AEs in both the groups will be analysed and compared; the details of each AE will be analysed.

## Discussion

In TCM, weakness in the “Liver” and “Kidney” and the invasion of “Wind” and “Damp” are considered the main causes of RA [14]. The meaning of “Liver”, “Kidney”, “Wind” and “Damp” in TCM is different from that in modern western medicine. Yishen Tongbi decoction contains *Fructus Ligustri Lucidi*, *Herba ecliptae*, *Eucommia ulmoides Oliver*, *Fructus lycii*, *Salvia miltiorrhiza Bge*, and *Tripterygium hypoglaucum (Levl.) Hutch*. Among these, *Tripterygium hypoglaucum (Levl.) Hutch* can dispel pathogenic wind and remove dampness in TCM, and has been widely reported to have a good effect in the treatment of RA [15]. *Fructus Ligustri Lucidi*, *Herba ecliptae*, *Eucommia ulmoides Oliver*, and *Fructus lycii* can nourish the liver and kidney [16–19]; *Salvia miltiorrhiza Bge* can promote blood circulation for removing blood stasis [20].

Data from animal and in vitro experiments indicate that *Fructus Ligustri Lucidi* improves bone metabolism and bone mass, and various individual compounds isolated from this plant reportedly have antiosteoporotic effects [21]. *Herba ecliptae* and its active ingredient, wedelolactone, inhibit the proliferation and differentiation of RAW264.7 osteoclast cells at low doses, but show cytotoxic effects on bone marrow stromal cells at high doses. These results suggest that *Herba ecliptae* prostrata and wedelolactone may possibly be used in the treatment of osteoporosis [22]. The 70% ethanol extract of *Eucommia ulmoides Oliver* inhibits the proliferation of synoviocytes, increases anti-inflammatory cytokine activity, inhibits pro-inflammatory cytokines, and reduces the degradation of cartilage and bone. These effects contribute to its symptom-alleviating effect in RA [23]. Because of the mixture of active polysaccharides of *Fructus Lycii*, *Lycium barbarum* has various effects, including antiosteoporotic, anti-inflammatory, and immunomodulatory effects in RA [24]. *Salvia miltiorrhiza Bge* is capable of anti-inflammatory and antiallergic effects, which have immunosuppressive activities [25]. Many components in *Tripterygium hypoglaucum (Levl.) Hutch* have been shown to have anti-inflammatory and immunosuppressive effects [26–29]. The components of Yishen Tongbi decoction have been demonstrated to be beneficial for RA by modern medical research. The composition and action of each herb is summarized in Table 1.

**Table 1:** Composition and action of Yishen Tongbi decoction

Ingredient	Action (TCM)	Pharmacological effect
*Fructus Ligustri Lucidi* (*nvzhenzi*)	Nourishing the liver and kidney	Improving bone metabolism and bone quality Antiosteoporotic
*Herba ecliptae* (*mohanlian*)	Nourishing the liver and kidney	Inhibiting osteoclast proliferation and differentiation
*Eucommia ulmoides Oliver* (*duzhong*)	Nourishing the liver and kidney Strengthening tendons and bones Preventing miscarriage	Suppressing the proliferation of synoviocytes Increasing the anti-inflammatory effects of interleukin-10 Inhibiting the serum and tissue levels of key pro-inflammatory cytokines Reducing the degradation of cartilages and bones
*Fructus lycii* (*gouqizi*)	Nourishing the liver and kidney Strengthening and nourishing marrow and essence Improving eyesight	Antiosteoporotic Anti-inflammatory Immunomodulatory
*Salvia miltiorrhiza Bge* (*danshen*)	Promoting blood circulation for removing blood stasis Clearing heart fire Tranquilization method	Anti-inflammatory Antiallergic Immunosuppressive
*Tripterygium hypoglaucum (Levl.) Hutch* (*kunmingshanhaitang*)	Dispelling pathogenic wind and removing dampness Relieving rigidity of muscles and activating collaterals	Anti-inflammatory Immunosuppressive

The content in parenthese is the Chinese spelling

*TCM* traditional Chinese medicine

Therefore, according to the theory of TCM and modern western medical research, Yishen Tongbi decoction should have a beneficial and curative effect on RA. Yishen Tongbi decoction has demonstrated a significant effect in clinical observations and studies on animal models of RA. This trial will evaluate whether Yishen Tongbi decoction has a definite therapeutic effect and whether the effect is greater than that of MTX.

Our research also has some limitations. First, there are some subjective indicators in the study that are evaluated by patients and physicians. Thus, these results may be influenced by their preferences. We can only assume that the physicians maintain a relatively uniform standard for evaluation, with patients needing further teaching on how to evaluate the disease situation correctly. Secondly, patients may have difficulty in adhering to the 24-week study period because of various reasons, and thus we need to strengthen the follow-up and communicate with patients in time to minimize the drop-out rate. The clinical trial is currently in progress. On completion of the trial, we hope to publish a definitive conclusion on the efficacy and safety of Yishen Tongbi decoction.

## Trial status

We are currently recruiting participants. The latest protocol version is version 3, 10 April 2019. The first participant was recruited on 6 August 2019, and the recruitment is expected to be completed in March 2021.

## Supplementary information

**Additional file 1.** SPIRIT 2013 checklist: recommended items to address in a clinical trial protocol and related documents.

## Data Availability

We declared that materials described in the manuscript, including all relevant raw data, will be freely available to any scientist wishing to use them for noncommercial purposes without breaching participant confidentiality.

## Abbreviations

ACR: American College of Rheumatology
AE: Adverse event
CDAI: Clinical Disease Activity Index
CRF: Case report form
DMARD: Disease-modifying antirheumatic drug
MTX: Methotrexate
RA: Rheumatoid arthritis
SDAI: Simplified Disease Activity Index
TCM: Traditional Chinese medicine
